# Drivers of mobile commerce adoption intention by Saudi SMEs during the COVID-19 pandemic

**DOI:** 10.1186/s43093-023-00190-8

**Published:** 2023-03-25

**Authors:** Thamir Hamad Alaskar, Amin K. Alsadi

**Affiliations:** Business Administration Department, Imam Mohammad Ibn Saud Islamic University, Riyadh, Saudi Arabia

**Keywords:** Critical drivers, Mobile commerce, Technology adoption, SMEs, Saudi Arabia

## Abstract

Grounded in the technology-organization-environment framework, this paper investigates critical drivers of mobile commerce (m-commerce) adoption intention by SMEs in Saudi Arabia, a developing country in transition, during the COVID-19 pandemic. A quantitative approach was adopted in this study for data collection and analysis. A proposed research model was tested and validated using PLS-SEM on data collected using a structured questionnaire from a sample of 171 Saudi SMEs located in the capital city of Riyadh. The findings reveal that top management support, environmental uncertainty and perceived benefits are the critical drivers of m-commerce adoption intention. However, perceived costs do not influence firms’ intentions to adopt m-commerce. This study contributes to a better understanding of m-commerce adoption intention in developing countries, particularly in Saudi Arabia. Both theoretical and practical implications of interest can be derived from this study.

## Introduction

Interest in and the use of mobile commerce within businesses has grown at a tremendous pace during the COVID-19 pandemic [73, 90]. M-commerce is the business of buying and selling goods and services through wireless handheld devices [22], and it has been described as one of the main submodels [17] or a considerable extension [8] of electronic commerce (e-commerce).

Small and medium enterprises (SMEs) account for the majority of firms in most countries; they are a very important part of modern economies, and their contribution to economic development is globally recognized [18]. Within the context of the Saudi economy, SMEs contribute to promoting economic growth, creating job opportunities, encouraging innovation and diversifying the economy into non-oil sectors.

One of the key objectives of the Saudi Vision 2030, which was launched in April 2016, is to increase the level of SMEs’ contribution to the nation’s GDP. Specifically, the target refers to increasing the contribution of SMEs from 20 percent of the GDP to 35 percent by 2030 [50]. To transform the SME sector into an engine for economic growth, the Kingdom has taken great interest in SMEs by improving the economic environment, increasing financing support programs, and enhancing regulatory frameworks. Moreover, the COVID-19 pandemic has demonstrated how digital innovation can enhance business resilience, and since Saudi Arabia has undergone a considerable digital transformation, SMEs have been encouraged to capitalize on Saudi Arabia’s digital revolution to access new markets and reduce their operational costs [1].

Previous research shows that digital technologies provide a platform for SMEs to reach new stages of development, improve efficiency and productivity, and gain competitive advantage [11, 80]. In particular, mobile technologies have several characteristics, including mobility, interactivity, ubiquity, localization and personalization, which set them apart from other technologies such as computers [54, 78]. These characteristics provide businesses, especially small and medium enterprises (SMEs), with numerous benefits, including the ability to directly and effectively sell to and serve customers, thereby significantly reducing operational costs and breaking the limitations of time and place [26, 97].

Despite the benefits that m-commerce could bring to SMEs and the economy, most of the existing studies focus on large enterprises [22]. Compared to large enterprises, the manager/owner represents the top management within the context of SMEs, and he or she acts as the main decision-maker, including on decisions to adopt new technological innovations such as m-commerce [72]. SMEs may also lack sufficient resources or be unwilling to commit a large portion of their resources to m-commerce adoption primarily because they are concerned about the costs of adoption, such as the infrastructure, training, and maintenance costs [21].

Moreover, according to [22], most of the previous research has examined m-commerce adoption in developed countries, while limited research has been conducted on SMEs within the special contexts of developing countries. Due to the differences in social, economic, cultural, legal and political contexts, it is difficult to generalize the research findings from developed countries to developing countries.

The current paper addresses the previous research gaps by seeking to better understand the m-commerce adoption of SMEs in Saudi Arabia which consider as one of the world's fastest-growing economies. Using a sample of Saudi SMEs and grounded on the technology-organization- environment framework (TOE), this study aims to address the following main research question: what are the impacts of perceived benefits and perceived costs, top management support and environmental uncertainty on the intentions of Saudi SMEs to adopt m-commerce? The following are the main objectives of this study:

1. To examine the effect of top management support as an organizational factor on the adoption of M-commerce among Saudi SMEs.
2. To assess the impact of perceived benefits and perceived costs as technological factors on the adoption of M-commerce among Saudi SMEs.
3. To evaluate the influence of environmental uncertainty as an environmental factor on the adoption of M-commerce among Saudi SMEs.

The rest of the paper is structured as follows: Sect. "Theoretical Background, Research Model, and Hypothesis Development" provides the theoretical background, research model, and hypothesis development. Sect. "Research Methodology" details and explains the research methodology. Sect. "Results" presents the empirical results. In Sect. "Discussion", the results are discussed. Finally, in Sect. "Conclusion, implications, and limitations", the paper concludes with conclusions, theoretical and practical implications, and limitations of the research.

## Theoretical background, research model, and hypothesis development

### TOE and organizational adoption of mobile commerce

The extant literature shows that the TOE framework is one of the most frequently used theories in technology adoption research [20]. Developed by Tornatzky and Fleischer [94], the TOE framework is an organizational-level theory that describes how a firm’s various contexts influence its adoption and implementation of technological innovations (van den [32, 96]. According to this framework, firms’ contexts are categorized into three categories: (1) the technological context is related to the characteristics of technologies in SMEs, (2) the organizational context is related to the characteristics of an organization in the adoption of innovation, and finally, (3) the environmental context constitutes the arena in which adopting organizations conduct their business [4, 21, 22].

Compared to other theories, the TOE framework is described as a dynamic and robust framework as it takes into account multiple contexts, thus providing a more comprehensive explanation of the adoption of a new technology than other theories that focus on a single context [72]. For example, the TEO framework places emphasis on the environmental context, which is predominantly overlooked by the Innovation Diffusion Theory (IDT) [58, 66].

The TOE framework is, also, considered a solid theoretical basis and a well-defined analytical framework, backed with strong empirical basis to study the phenomena of technology adoption [20, 22]. As a result, this framework has been widely adopted in previous empirical studies, either as the sole framework or combined with other theories, to identify the critical drivers of the adoption and assimilation of various types of technological innovations, such as e-business [66, 71], RFID [47, 103], ERP [16, 95], cloud computing [32, 56], BDA [10, 51] and mobile commerce [11, 76, 79].

However, despite this wide employment of the TOE framework to study the adoption of various technological innovations, many researchers have noted that the specific factors identified within the three contexts (technological, organizational, and environmental) vary across different industries and technologies; moreover, the findings for each given factor are inconsistent among previous research [20, 67]. Thus, the results of prior research call for the need to develop an adoption model for each specific technology/industry [66, 101].

In the case of Saudi Arabia, and in line with Vision 2030, the government of Saudi Arabia is actively promoting the adoption of online trade technologies among businesses, with a specific emphasis on SMEs [12]. The country's digital infrastructure is relatively advanced and is developing rapidly compared to other markets. Over the last few years, the country has made significant strides in terms of internet connectivity and coverage, and it is currently leading among fifth-generation (5G) countries in terms of network coverage and speed [60], Mordor [63].

All the previous features have made M-commerce more popular among users, the number of mobile phone internet users in Saudi Arabia in 2022 is estimated at 33.14 million which represents 92.46% internet penetration of the total population [87, 88] and the average resident in Saudi Arabia owns more than one mobile phone. Saudi Arabia is viewed as a large market for online shopping [39] and is well-positioned to become a leading player in e-commerce industry within the region [60]. Both private and government organizations are working toward increasing availability of mobile applications in the marketplace, which has contributed to the growing popularity of m-commerce among users [104].

Despite these advantages, many studies and reports show that few commercial organizations in Saudi Arabia adopted online trade technologies, particularly, among SMEs (e.g., [7, 11, 15, 27, 68].

M-commerce, as defined earlier, refers to the buying and selling of goods and services through mobile devices. In recent years, m-commerce has gained significant attention and importance within the context of Saudi Arabia. However, despite the growing interest in m-commerce in Saudi Arabia, there is a scarcity of research that focuses on this topic and examines the factors that influence the adoption of m-commerce in SMEs at the organizational level.

The majority of the existing studies on m-commerce adoption in Saudi Arabia focus on the perspective of customers or users, for example, Aldhaban [5], Gull et al. [39], and Wasiq et al. [104]. These studies examined how consumers use m-commerce, rather than the factors that influence organizations to adopt m-commerce in their business operations. There is a lack of research that looks at the organizational level determinants of m-commerce adoption in Saudi SMEs.

To date, we were able to find only one qualitative study that has examined m-commerce adoption at the organizational level in Saudi Arabia. Alfahl et al. [6] conducted an exploratory study to identify the main factors that influence the adoption of m-commerce in 15 large organizations from the banking and telecommunications sectors in Saudi Arabia. The study revealed that security, organizational policy, and ICT infrastructure significantly affect the organizational adoption of m-commerce. However, it is important to note that the study targeted large organizations, not SMEs, and it was conducted in 2013, when many conditions such as telecommunications infrastructure and mobile technologies were less developed than they are today. As a result, the findings of the study may not be as relevant to the current state of m-commerce adoption among SMEs in Saudi Arabia, as the conditions have greatly changed.

In summary, the previous discussions clearly show that multiple conditions are becoming more supportive toward the adoption of m-commerce SMEs within the special context of Saudi Arabia as one of the world's fastest-growing economies (such as the favorable customers preferences, the support from both government and private organizations and the fairly developed digital infrastructure). This implies that the adoption decision might be dependent upon other organizational, technological, and environmental factors, mainly, the degree of the top management support, the perceptions of whether the benefits of adopting m-commerce outweighs its costs, and the degree of uncertainty in the environment. Hence, grounded on the TOE framework, a research model was developed in the current study to assess and verify the impacts of these factors on the intention to adopt M-commerce among Saudi SMEs.”

**Fig. 1:**
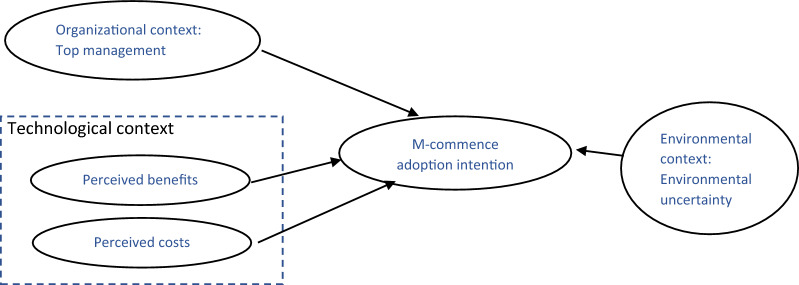
Research model

As depicted in Fig. 1, our research model includes perceived benefits and perceived costs under the technological context, top management support under the organizational context, and environmental uncertainty under the environmental context. Each of these drivers is discussed in the following sections.

### Organizational context: top management support

Top management support involves top managers’ level of awareness, commitment, and engagement toward introducing technological innovation (e.g., m-commerce) [33, 46, 77, 98]. This support is expressed in the mission of the business, the willingness to take risks and the establishment of a supportive organizational environment for the technological adoption [10, 22].

The findings of previous empirical research on technological innovation adoption show that top management support is one of the critical success factors, as it provides firms with the required strategic direction, authority, resources, and supportive environment during the adoption process [4, 89].

However, the literature suggests that the top management support of m-commerce adoption is more important for SMEs than for larger enterprises. This is because the owner/entrepreneur “is at center of his or her organization and the full driver of all business decisions” [31], p. 5), including the adoption of new technologies. Moreover, Hameed et al. [43] contend that “top management support in allocating sufficient resources for adoption is overshadowed by lack of resources in small organizations that contributed more to the adoption of IT”. Thus, we hypothesize the following:

**H1:** Top management support is positively related to the intention to adopt m-commerce.

### Technological context: perceived benefits and perceived costs

#### Perceived benefits

The perceived benefits concept has been defined as the expected benefits of adopting new technology [26]. Alaskar et al. [4] suggest that other terms with the same meaning can also be used, such as “expected benefits” or “relative advantage”. Otieno et al. [65] explain that it is critical for every organization to calculate the costs/benefits of implementing new technology. Chau et al. [22] argue that the main perceived benefits that could be gained from implementing new technology include improving products and profitability, attracting new customers, enhancing new markets, gaining competitive advantages and reducing costs by integrating main business activities such as order and payment processes with after-sales services. They further argue that m-commerce implementation can help firms increase performance by allowing them to complete the reengineer business processes phase.

Grandhi and Wibowo [37] and Picoto et al. [70] mention that the features that enhance user identification through exact location, portability, and immediate connectivity and that are mainly related to mobile technologies allow firms to support their internal activities and add value. In addition, Grandhi and Wibowo [37] further argue that although the benefits of m-commerce are not certain, firms are willing to adopt this new technology because it can impact the firm management approach and behavior style, as mentioned by Lee [53]. In addition, m-commerce can be used as a critical tool for universal competition, especially for small and medium firms, by illuminating the barrier to entering new markets [22]. Cedeno [19] suggest that once firms and sellers recognize that m-commerce will help to increase their sales and obtain access to new markets, they will start using the technology and try to achieve successful implementation.

In addition, Chau et al. [22] show that, for SMEs, perceived benefits are vital for m-commerce implementation, as mentioned in many previous studies. They further argue that the decision to implement new technologies is mainly based on comparing risks and benefits, as mentioned by Ghobakhloo and Tang [35]. Therefore, the intention to implement and use technology such as commerce is dependent on the level of perceived benefits [22]. Therefore, in this study, we emphasize the impact of perceived benefits and hypothesize as follows:

**H2:** Perceived benefits are positively related to the intention to adopt m-commerce.

#### Perceived cost

Perceived cost has been defined as the expense of technology once in use; this includes many aspects, such as preliminary purchase price and subscription, use cost, maintenance charges, and training of firm members in technology use [31], [36]. Tan et al. [91] and Chau et al. [22] explain that perceived cost is a particularly important consideration for SMEs located in developing countries, as financial constraints in those firms play a critical role in the decision to adopt technology. Chau et al. [22] further argue that the steps of technology implementation are mainly associated with cost, so high cost may lead to slow implementation processes, as mentioned by Alam et al. [3]. Rind et al. [75] show that aspects such as access and transaction costs can play an important role in the m-commerce user experience, which may impact usage of the technology.

On the other hand, Sila [84] and Yadav et al. [106] argue that perceived cost is less important and does not have a positive impact on m-commerce implementation. Furthermore, Rind et al. [75] explain that many studies on m-commerce show that there is a negative relation between perceived cost and consumers’ use intention. However, Cedeno [19] mentions that the cost related to m-commerce adoption must be at its lowest, such as transactions and maintenance costs. He further argues that sellers prefer to adopt m-commerce that offers no cost, although there are benefits in future. In addition, Chau et al. [22] emphasize that cost importance is fully related to some technologies, as mentioned by many prior studies [13, 59, 91]. Therefore, in this study, we stress the impact of perceived cost and hypothesize as follows:

**H3:** Perceived cost is positively related to the intention to adopt m-commerce.

### Environmental context: environmental uncertainty

The business environment can be characterized as turbulent or stable, depending on its uncertainty rates. Environmental uncertainty refers to turbulence or changes “in customer markets, consumer preferences, technologies, or competitive intensity” [86]: p. 2031). The current study focuses on two critical dimensions of environmental uncertainty: environmental dynamism and environmental complexity [55, 82].

Environmental complexity is defined as ‘the heterogeneity and range of an industry and/or an organization’s activities’ [100], p. 127). Organizations operating in complex environments have to interact with highly diverse customer buying habits, competitive nature, and product lines, as such, this environment is especially challenging for managers to understand and navigate [23, 55].

Environmental dynamism is also a challenging aspect of environmental uncertainty; it refers to the rate and unpredictability of environmental changes. The literature shows that these changes include technological changes, unpredictable competitors' moves, and changes in customer demand [23, 55, 64].

Extant research shows that IT innovations are more related to a turbulent environment than a stable one [102]. Coping with an uncertain environment creates the need for larger amounts of information and greater information processing and sharing, thus, managers are forced to recognize the importance of developing their firms’ IT capability and integrating it into their business planning [49, 52].

Moreover, organizations operating in complex and dynamic environments are susceptible to various ideas from their markets and are more incentivized to distinguish themselves from their competitors by adopting and investing in new technologies such as m-commerce [14, 34, 74, 83].

Based on the previous discussion, we contend that the increase in environmental uncertainty both locally and globally will encourage Saudi firms to adopt m-commerce to expand their markets and improve their competitiveness. Thus, we propose the following:

**H4**: Environmental uncertainty is positively related to the intention to adopt m-commerce.

## Research methodology

This study used the survey method with a questionnaire that was based on constructs identified from previous studies. All constructs were measured using items adapted from previous research. The research variables, measurement items and related studies are presented in Table 1. All measurement items were scored on a five-point Likert scale ranging from 1 “Strongly disagree” to 5 “Strongly agree.

**Table 1:** Variables, Items and Related studies

Variables (related studies)	Items
M-commerce adoption [25]	Our firm intends to adopt M-commerce Our firm has a certain plan for M-commerce adoption Our firm has a strong commitment to adopt M-commerce
Environmental uncertainty [23, 64]	The product/services technologies in our industry change very quickly Products and services in our industry become obsolete very quickly We can predict what our competitors will do next We can predict when our products/services will demand changes In our industry, there is considerable diversity in customer buying habits In our industry, there is considerable diversity in the nature of competition In our industry, there is considerable diversity in product lines
Top management support [32, 56, 103]	Top management has generally been likely to take risks involved in the adoption of the M-commerce Top management is likely to consider the adoption of M-commerce which is strategically important Top management have policies that encourages usage of M-commerce Top management have strong positive views on how M-commerce could transform business
Perceived Benefits [22, 81, 93] Zhu et al. (2006)	Operating cost saving Simplification of the operating procedures Increase in market share Growth of revenue Creation of marketing channels Improvement of the company’s image Improvement in competitiveness Enhancement of customer services
Perceived cost [9]	The cost of the new mobile technologies is very high The amount of money and time invested in training employees to use the adopted new technologies is very high The cost of maintenance and support of the new mobile technologies is very high for our business The telecommunications (bandwidth) cost is very high

The data for this study were collected during the peak of the COVID-19 pandemic, which presented significant challenges due to multiple lockdowns and safety measures. To overcome these difficulties, the researchers used convenience and purposive sampling techniques to target the respondents. The questionnaire was directly delivered or mailed to SMEs based in Riyadh, the capital city of Saudi Arabia. Riyadh was chosen as the location for the study as it is the largest city in Saudi Arabia and home to many successful SMEs across a range of industries [62]. The targeted participants in the study are considered key decision-makers and informants when studying technology adoption, such as CIO, E-commerce managers, project managers, and IT managers [49, 61].

In total, the data were gathered from a sample size of 185 and a total of 171 usable responses, which represents a 92% response rate. The sample characteristics are illustrated in Table 2. In addition, this study used the partial least squares structural equation modeling (PLS-SEM) technique from Smart PLS V 3.3.7 [28] software to test the hypotheses and model. Compared to CB-SEM, PLS-SEM is a useful technique for determining the most significant variables in predicting target constructs, as it does not require normal distribution assumptions [42, 48]. Furthermore, it has been found to be more robust when used in the analysis of small sample sizes [51, 85].

**Table 2:** Characteristics of the sample

Sector	No	%	Employees	No	%
Manufacturing	47	27.4%	Less than 50	53	30.9%
Wholesale and retail	60	35.1%	50–249	79	46.1%
Healthcare	18	10.5%	250–499	39	22.8%
			Respondent’s position		
Insurance	16	9.3%	CIO	18	10.5%
			IT Manager	29	16.9%
Others	30	17.5%	Project manager	47	27.4%
			E-commerce Manager	10	5.8%
			System Analyst	18	10.5%
			E-commerce specialist	20	11.6%
			Other	29	16.9%

## Results

### Measurement model

Regarding reliability and internal consistency, Table 3 shows that the values are higher than 0.7 for composite reliability and 0.6 for Cronbach’s alpha, which refer to acceptable values, as mentioned by Hair et al. [40] and Griethuijsen et al. [38], respectively. In addition, Table 3 shows that the lowest satisfactory value for the AVE index exceeds 0.5, and all values show an acceptable level of discriminant validity for the items used when associated with the squared correlations, as determined by Hair et al. [41]. Generally, and based on the values shown in Tables 3 and 4, all attained values are within a satisfactory range, which allows us to continue to the next step to examine the research hypotheses.

**Table 3:** Loadings, Cronbach's alpha, rho_A, composite reliability, average variance extracted (AVE)

Constructs	Items	Loadings	Cronbach's Alpha	rho_A	Composite Reliability	Average Variance Extracted (AVE)
Environmental uncertainty	﻿Env1	0.776	0.762	0.772	0.840	0.513
	﻿Env2	0.665				
	﻿Env3	0.678				
	﻿Env4	0.694				
	Env5	0.760				
M-commerce adoption	Mco.Ado1	0.850	0.849	1.050	0.905	0.762
	Mco.Ado2	0.954				
	Mco.Ado3	0.808				
Perceived benefits	Per.Ben1	0.736	0.768	0.771	0.843	0.518
	Per.Ben2	0.737				
	Per.Ben3	0.724				
	Per.Ben4	0.684				
	Per.Ben5	0.716				
Perceived cost	Per.Cos1	0.811	0.791	0.810	0.861	0.608
	Per.Cos2	0.760				
	Per.Cos3	0.786				
	Per.Cos4	0.760				
Top management support	Top.Sup1	0.752	0.698	0.708	0.832	0.624
	Top.Sup2	0.838				
	Top.Sup3	0.776

**Table 4:** Correlation matrix

	Environmental Uncertainty	M Commerce Adoption	Perceived Benefits	Perceived Cost	Top Management Support
Environmental uncertainty	0.716				
M-commerce adoption	0.513	0.873			
Perceived benefits	0.503	0.482	0.720		
Perceived cost	0.272	0.331	0.205	0.780	
Top management support	0.520	0.662	0.469	0.346	0.790

To assess the presence of common method bias, Harman's single-factor test was examined using SPSS. An exploratory factor analysis was performed with no rotation, and the results revealed that the first factor explained (27.619%) less than 50% of variance [44, 69]. This indicates that common method bias is not an issue in the current study. In addition, the results of the Heterotrait-Monotrait Ratio (HTMT) presented in Table 5 indicate that all values fall below the threshold of 0.9 as recommended by Hair et al. [40] and Henseler et al. [45]. Thus, discriminant validity is also demonstrated.

**Table 5:** Validity tests (Heterotrait-Monotrait Ratio (HTMT))

Constructs	Environmental uncertainty	M commerce adoption	Perceived benefits	Perceived cost	TMS
Environmental uncertainty					
M commerce adoption	0.606				
Perceived benefits	0.654	0.594			
Perceived cost	0.333	0.384	0.260		
TMS	0.701	0.853	0.639	0.455	

### Structural model

The structural model using the SEM-PLS module integrated into SmartPLS was used to complete the estimation process of the structural model. The structural analyses address the strength of the structural path, and the joint productivity showed that the R2 value of the dependent variable is suitable at 0.508 for m-commerce adoption, as shown in Table 6. These values imply a satisfactory productivity value, which indicates that the dependent variables are well explained by the independent variables; the minimum acceptable value of the R2 index should exceed 0.1, as stated by Falk and Miller [30].

**Table 6:** R^2^ and global fit indices

	R^2^	Average Variance Extracted (AVE)
Environmental uncertainty	–	0.513
M commerce adoption	0.503	0.762
Perceived benefits	–	0.518
Perceived cost	–	0.608
Top management support	–	0.624
Average	0.503	0.605
AVE * R^2^	0.304	
GoF		0.551

Furthermore, the global criterion of the GOF index has been established for validating PLS models and assessing the overall prediction, as it is considered the main index in both the measurement model and structural model [92, 99]. Table 6 shows that the indices are statistically fit with an overall goodness-of-fit index of 0.551, as the satisfactory cutoff value of the GoF is 0.5, as mentioned by Wetzels et al. [105].

**Fig. 2:**
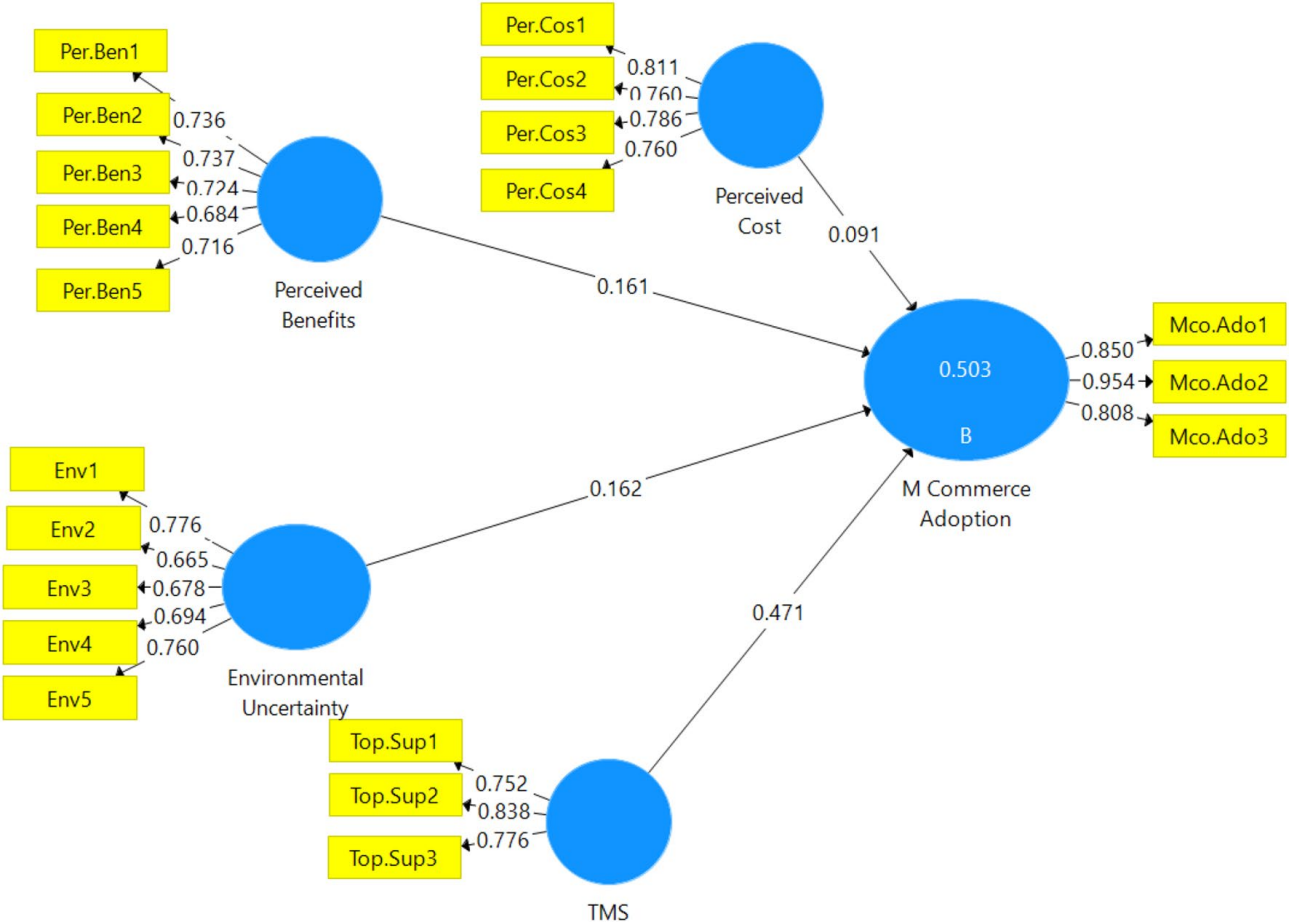
Fitted model

Table 7 shows that the direct impact of perceived cost is not significant at the 5% level. Based on these findings, it is possible to state that Hypothesis H3 is not supported, while all other hypotheses are supported. The next section will discuss the results shown in this section (Fig. 2).

**Table 7:** Summary results of the hypothesis development

Constructs	Original Sample (O)	Sample Mean (M)	Standard Deviation (STDEV)	T Statistics (|O/STDEV|)	P Values
Environmental Uncertainty—> M-commerce Adoption	0.162	0.167	0.074	2.186	*0.029
Perceived Benefits—> M-commerce Adoption	0.161	0.160	0.060	2.686	*0.007
Perceived Cost—> M-commerce Adoption	0.091	0.097	0.063	1.460	0.145
Top Management Support—> M-commerce Adoption	0.471	0.472	0.068	6.937	*0.000

## Discussion

The findings of the current study show top management support as the most important driving factor for the intention to adopt mobile commerce among Saudi SMEs. The importance of top management support is consistent with the findings of previous studies [31, 54,65]. This can be explained by the dominant role of top management, particularly in SMEs, where top management generally represents the owner and sole decision-maker whose support and involvement contribute to the success of the m-commerce adoption process, especially at the early stages.

Environmental uncertainty was also found to be a significant facilitator of mobile commerce adoption by Saudi SMEs. This finding is consistent with the findings of other studies related to innovation adoption, such as big data [2] and ERP [57]. The current study was conducted during the COVID-19 pandemic; this pandemic has dramatically increased the levels of environmental uncertainty within both the Saudi and international markets, which, in turn, has fostered SMEs’ intention to adopt mobile commerce to keep their everyday activities running. In particular, the associated safety measures that were applied during the pandemic, such as lockdowns and social distancing, forced firms to close their doors and customers to stay in their homes, therefore, mobile phones were the primary interaction platform among SMEs’ employees, customers, and other trading partners.

Regarding technological factors, this study shows that perceived benefits would positively impact firms’ intention to adopt mobile commerce. This result implies that Saudi SMEs are becoming more aware of the potential benefits of mobile commerce, which include lowering costs and simplifying procedures, increasing market share and revenues, and improving marketing channels, image, competitiveness, and customer services. The increased perception of these benefits that m-commerce could bring to Saudi SMEs has positively affected their adoption intention to exploit new opportunities and survive in today’s uncertain market environment. This result is also in line with the findings of some previous studies, such as [24, 31, 37, 65].

Contrary to perceived benefits, our findings show that perceived costs are not supported as a determinant of the intention to adopt mobile commerce; this finding is inconsistent with the findings of study by Fatoki [31]. This outcome could be explained by multiple reasons. First, the costs for mobile commerce adoption have recently decreased, which allows for its adoption by many SMEs with limited resources. Second, the intensity of competition within the context of the Saudi market has dramatically increased due the growing number of competitors and the efforts of firms to meet customers’ preferences for online shopping options. Finally, the previously discussed consequences of the COVID-19 pandemic led to a condition in which the perceived benefits of adoption exceeded its perceived costs, which urged Saudi SMEs to transition to a mobile commerce business model to improve their sustainability and competitiveness during the crisis.

## Conclusion, implications, and limitations

This study investigated some critical drivers of the intention of Saudi SMEs to adopt m-commerce. From a theoretical perspective, the current study developed a predictive model for the adoption of m-commerce by SMEs in Saudi Arabia which consider as a one of the world's fastest-growing economies, and it also verified the applicability of the TOE framework in understanding mobile commerce adoption in such a context. Moreover, this study examined environmental uncertainty, which is an environmental factor that has been little explored in prior research yet has increased dramatically during recent years due to multiple events, including the COVID-19 pandemic and the Russian-Ukraine conflict. As such, this study shows how environmental uncertainty has played a major role during recent years in pushing Saudi SMEs to consider the adoption of mobile commerce as a competitive necessity rather than a competitive advantage.

From a practical perspective, top managers of Saudi SMEs should be aware that their vital support for the adoption of mobile commerce must be translated into action by making the required decisions to provide adequate resources and create a positive environment for the adoption process. They should, also, consider mobile commerce as a key strategy for addressing challenges and seizing new opportunities presented by today's high environmental uncertainty to stay competitive. This perspective on mobile commerce can assist SMEs in better understanding and adapting to changing market conditions and customer preferences, expanding their reach to new customers, reducing costs, and strengthening customer loyalty through improved interactions and relationships. This study also concludes that positively perceived benefits are the best predictors of Saudi SMEs’ intentions to adopt mobile commerce, while negatively perceived costs do not seem significant. Furthermore, this study contributes to the existing body of literature on technology adoption, specifically in the context of mobile commerce and SMEs within developing countries. It provides further evidence that perceived benefits, rather than perceived costs, are a key driver of technology adoption decisions. Moreover, the findings suggest that if SMEs can be made aware of the potential benefits of mobile commerce, such as increased sales and cost savings, they will be more likely to adopt m-commerce.

Governmental agencies, such as the Small and Medium Enterprises General Authority (Monsha'at) and the Saudi Arabian General Investment Authority (SAGIA), can play a crucial role in promoting the adoption of m-commerce among SMEs by communicating the benefits of m-commerce, providing resources, and training to SMEs owners and employees. Different incentives such as tax incentives and subsidies can also be implemented to encourage SMEs to adopt m-commerce. Additionally, financial and technical assistance can be provided to alleviate the costs associated with m-commerce adoption, such as investments in hardware and software. Environmental uncertainty can be mitigated by promoting a stable legal and economic environment and providing clear regulations and guidelines for m-commerce adoption.

Despite the contributions of the current study, there are some limitations that could provide avenues for future research. First, based on the TOE framework, only a small set of organizational, technological, and environmental factors were considered in the current research model. Future research could consider combining other theoretical perspectives to study more factors. We also suggest studying the mediating or moderating effects of some environmental factors, such as competitive pressure and governmental support, to obtain a deeper understanding of mobile commerce adoption in SMEs. Second, we conducted our sampling in Riyadh, the capital of and the largest city in Saudi Arabia; thus, it is impossible to extend the findings to SMEs located in other geographic regions of Saudi Arabia. Future research should consider a larger national sample. Furthermore, this study revealed that environmental uncertainty plays a crucial role in fostering SMEs' intentions to adopt mobile commerce technology. However, empirical studies that have examined the role of mobile commerce in highly uncertain environments are still scarce. Future research could benefit from conducting qualitative studies to gain a deeper understanding of how and what mobile commerce strategies and tactics can make SMEs more resilient in today's highly diverse and unpredictable market conditions.

Finally, the current study focused on examining the intention to adopt m-commerce as an independent variable. Future research may consider examining the actual adoption and implementation of m-commerce in Saudi SMEs as a dependent variable and its impacts on other aspects of SMEs, such as operational, market and financial performance indicators, as independent variables.

## Data Availability

The datasets used during the current study are available from the corresponding author on reasonable request.

## Abbreviations

TOE: Technology-organization-environment framework
M-commerce: Mobile commerce
SMEs: Small and Medium Enterprises
Mco.Ado: Mobile commerce adoption
Per.Ben: Perceived benefits
Per.Cos: Perceived cost
Top.Sup: Top management support
Env.: Environmental uncertainty
PLS-SEM: Partial least squares structural equation modeling
